# Myocardial infarction, atrial fibrillation and mortality: timing is everything

**DOI:** 10.1007/s12471-015-0710-9

**Published:** 2015-06-05

**Authors:** N.W.E. van den Berg, J.R. de Groot

**Affiliations:** Heart Center, Department of Cardiology, Academic Medical Center/University of Amsterdam, Meibergdreef 9, 1105 AZ Amsterdam, The Netherlands

New-onset atrial fibrillation (AF) occurs commonly following myocardial infarction (MI) with an incidence ranging from 5 to 23 %. Unlike other complications of MI, such as cardiac failure or ventricular arrhythmias, it has long been underappreciated as a cause of adverse outcome [1, 2]. Indeed, several studies demonstrated an independent risk of AF on mortality, whereas others showed no significant independent effect, which may suggest that the simultaneous onset of AF and MI merely reflects the severity of the underlying disease. Interestingly, there are also reports of a beneficial effect of AF following MI on outcome [3–5]. Meta-analyses nevertheless consistently show that both pre-existing AF and new-onset AF following MI independently increase the risk of mortality by 40–50 %, but controversy remains whether this altered risk relates to the pre-existence and type of AF, MI presentation or performed intervention [1, 6]. In 2011, a meta-analysis including 43 studies of in total 278,854 patients demonstrated a 46 % excess in mortality in patients with AF after acute MI. A subgroup analysis of nine studies that explicitly defined the new onset of AF showed similar results with an odds ratio (OR) of 1.37 (95 % confidence interval: 1.26–1.49) for mortality [5]. In 2012, a second meta-analysis even reported an OR of 3.38 for patients with new-onset AF. Here, the risk of death was 87 % higher for patients with new-onset AF compared with those with (possible) pre-existing AF, suggesting that the risk of mortality depends on the timing of AF onset.[1]

In this issue of the *Netherlands Heart Journal*, Gal et al. provide an analysis of patients included in the On-TIME II study, in which patients undergoing primary percutaneous coronary intervention for ST-elevation MI (STEMI) were randomly assigned to tirofiban or placebo treatment on top of usual medical treatment [7, 8]. The authors confirm the relation between AF and mortality in this setting and report the 30-day mortality in 830 patients without a previous history of AF, stratified by the moment of AF onset: (1) AF occurrence on the day of admission for MI, during which participants were continuously monitored; (2) AF between 24 and 72 h after admission, with continuous monitoring for another 24 h (24–48 h) and a single electrocardiogram (ECG) the following day (48–72 h) and (3) AF > 72 h after admission, when ECGs were performed daily or whenever symptoms occurred. During follow-up, AF was detected in 41 patients on the day of admission, in 14 in the subsequent 48 h and in 18 in the last 27 days. This decreasing prevalence of AF may partially reflect the lower amount of AF monitoring. The incident of new-onset AF was significantly associated with 30-day mortality when AF occurred on the day of admission. Three patients (7.3 %) died in the AF group, versus 17 in the non-AF group (2.2 %, *p* = 0.036). When AF occurred between 24–72 h after admission, mortality was 14.3 and 1.4 % in the AF group and non-AF group, respectively (*p* < 0.001). There was no association between AF and mortality when AF was diagnosed > 72h after admission, as no deaths were seen in the new-onset AF patients, but > 1 % of the patients without AF died.

The suggestion presented by Gal et al. that early- but not late-onset AF after MI is associated with increased mortality is interesting. However, the higher mortality in patients with AF < 72 h after admission cannot exclusively be ascribed to the timing of AF onset. First, a daily ECG is clearly less sensitive than continuous rhythm monitoring during the first 48 h after admission. AF could therefore easily have been underdiagnosed during the second and especially the third period when mortality only occurred in patients presumed to be without AF. The investigators further did not adjust for the distinct differences at baseline between AF and non-AF patients regarding age, smoking, diabetes mellitus, history of MI and Killip classification. It is therefore well conceivable that AF is not truly independent as a risk factor in this cohort. Potentially, biomarkers of underlying pathological processes could be collected in patients and mark the mechanisms leading to both AF and death. Understanding this could provide us with tools to anticipate on the increased risk of mortality in these patients; as for now, it remains unclear how to prevent AF-related death after MI.

In the current study, Gal et al. for the first time demonstrate an association between early-onset AF after STEMI and mortality. The question remains unanswered whether the early onset of AF after MI should be regarded as an indicator of preset misery or as a causal factor leading to mortality. Until this issue is resolved, patients with early onset of AF after MI should be considered at increased risk, and where possible, extra care should be given to prevent a detrimental outcome.

## Funding

Dr De Groot is supported by a VIDI grant from NWO/ZonMW 016.146.310.

## Conflict of interest

None declared.

## References

[1] Angeli F, Reboldi G, Garofoli M (2012). Atrial fibrillation and mortality in patients with acute myocardial infarction: a systematic overview and meta-analysis. Curr Cardiol Rep.

[2] Goldberg RJ, Seeley D, Becker RC (1990). Impact of atrial fibrillation on the in-hospital and long-term survival of patients with acute myocardial infarction: a community-wide perspective. Am Heart J.

[3] Mehta RH, Dabbous OH, Granger CB (2003). Comparison of outcomes of patients with acute coronary syndromes with and without atrial fibrillation. Am J Cardiol.

[4] Pizzetti F, Turazza FM, Franzosi MG (2001). Incidence and prognostic significance of atrial fibrillation in acute myocardial infarction: the GISSI-3 data. Heart.

[5] Kytö V, Jussi S, Päivi R. (2015). Gender and in-hospital mortality of ST-Segment Elevation Myocardial Infarction (from a Multihospital Nationwide Registry Study of 31,689 Patients). Am J Cardiol.

[6] Jabre P, Roger VL, Murad MH (2011). Mortality associated with atrial fibrillation in patients with myocardial infarction: a systematic review and meta-analysis. Circulation.

[7] Gal P, Demirel F, Adiyaman A, et al. Prognostic significance of incident atrial fibrillation following STEMI depends on the timing of atrial fibrillation. Neth Heart J 2015;23: DOI: 10.1007/s12471_015_0709_2. 10.1007/s12471-015-0709-2 PMC4547948 26021618

[8] Van ’t Hof AW, Ten Berg J, Heestermans T (2008). Prehospital initiation of tirofiban in patients with ST-elevation myocardial infarction undergoing primary angioplasty (On-TIME 2): a multicentre, double-blind, randomised controlled trial. Lancet.

